# Impact of a midline catheter prioritization initiative on device utilization and central line-associated bloodstream infections at an urban safety-net community hospital

**DOI:** 10.1017/ash.2024.21

**Published:** 2024-02-16

**Authors:** Alfredo J. Mena Lora, Brenna Lindsey, Stephanie Echeverria, Mirza Ali, Candice Krill, Eden Takhsh, Susan C. Bleasdale

**Affiliations:** 1 Division of Infectious Diseases, Department of Medicine, University of Illinois at Chicago, Chicago, IL, USA; 2 Saint Anthony Hospital, Chicago, IL, USA

## Abstract

Overuse of peripherally inserted central catheters (PICCs) can lead to idle central line (CL) days and increased risk for CL-associated bloodstream infections (CLABSIs). We established a midline prioritization initiative at a safety-net community hospital. This initiative led to possible CLABSI avoidance and a decline in PICC use.

The use of peripherally inserted central catheters (PICCs) has substantially increased in past decades.^1,2^ PICCs offer advantages such as extended dwell times and reduced complications associated with insertion.^2^ However, PICCs often remain in use beyond their initial indication, leading to idle central line (CL) days and higher risk for CL-associated bloodstream infections (CLABSIs).^2,3^ When high-risk infusions or vasopressors are no longer necessary, alternative venous access methods should be considered. The midline catheter (ML) has emerged as a practical alternative, offering lower bloodstream infection (BSI) rates compared to PICCs, comparable dwell times, and reduced complications compared to CLs.^4^ We established a program that prioritized ML use in patients requiring venous access when peripheral intravenous access was not feasible. We seek to describe the impact of this ML prioritization initiative on device utilization and complications at an urban safety-net community hospital.

## Methods

### Study design and setting

We performed a quasi-experimental study to evaluate the impact of a ML prioritization initiative in a 151-bed safety-net community hospital. The preintervention period was from January 2018 through December 2018, and the postintervention period from January 2019 to December 2021.

The ML initiative was incorporated into a daily interdisciplinary safety huddle (DISH). DISH is a forum where hospital unit and department managers gather to discuss safety and infection control variables at 8 A.M., including isolation needs and device use. CL indications, duration, and removal plans, are reported at DISH. The infection preventionist (IP) reviews device indications and recommendations for removal are made when CLs are no longer needed.^2^ Unit managers follow up on these recommendations, and if issues arise, hospital administration helps resolve them within 24 hours. DISH led to a reduction in device utilization rates (DURs).^5^ Our ML prioritization initiative was established as a component of this comprehensive CLABSI prevention strategy.

### Intervention

CLs were reviewed daily at DISH to assess indications, such as total parenteral nutrition, hyperosmolar solutions, and vasopressors.^2,5^ If indications were not present and peripheral access not feasible, MLs were recommended rather than PICCs or CLs. New orders for PICCs were reviewed by IP. If no indications were present, the ordering provider was notified by the PICC nurse and MLs recommended if peripheral access was not feasible. If the ordering provider disagrees, the Medical Director of Infection Prevention or Chief Quality Officer would discuss the case with the provider and deliver just-in-time education. The provider would have final decision-making on device choices. Hospital-wide education on this policy and ML benefits were provided. Guidelines for selecting the appropriate venous access device were disseminated to new staff on orientation and to existing staff twice per year (Supplement A).

### Data collection and analysis

Data on device utilization of PICCs and MLs were collected and analyzed for the study period. Deep venous thrombi (DVTs), BSIs, and CLABSIs were reviewed and compared before and after our intervention. Descriptive statistics were used to summarize data and assess differences in utilization before and after the intervention. Primary outcome measures included device use, DVTs, and BSIs associated with device use. DUR and cost were secondary outcomes.^6^Potential cost savings from avoided CLABSIs were estimated by multiplying the number of ML BSIs that would have met National Healthcare Safety Network (NHSN) criteria by the estimated cost of one CLABSI ($48,108) and BSI ($20,000).^6,7^ Estimated savings were then determined by assessing the differential between these two costs.

## Results

### Device utilization trends

**Figure 1.**
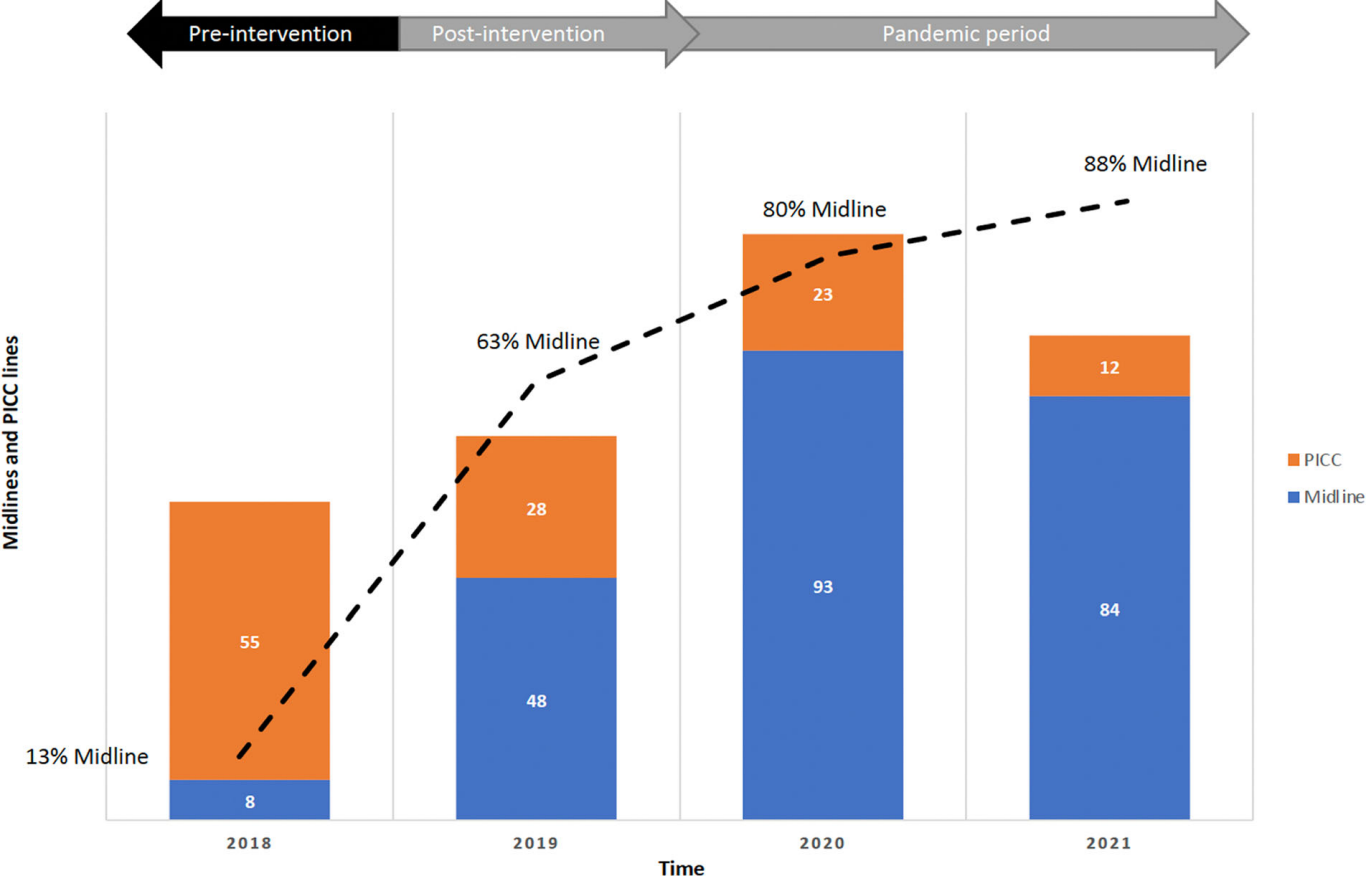
Midlines and PICC lines during our preintervention and postintervention period

**Figure 2.**
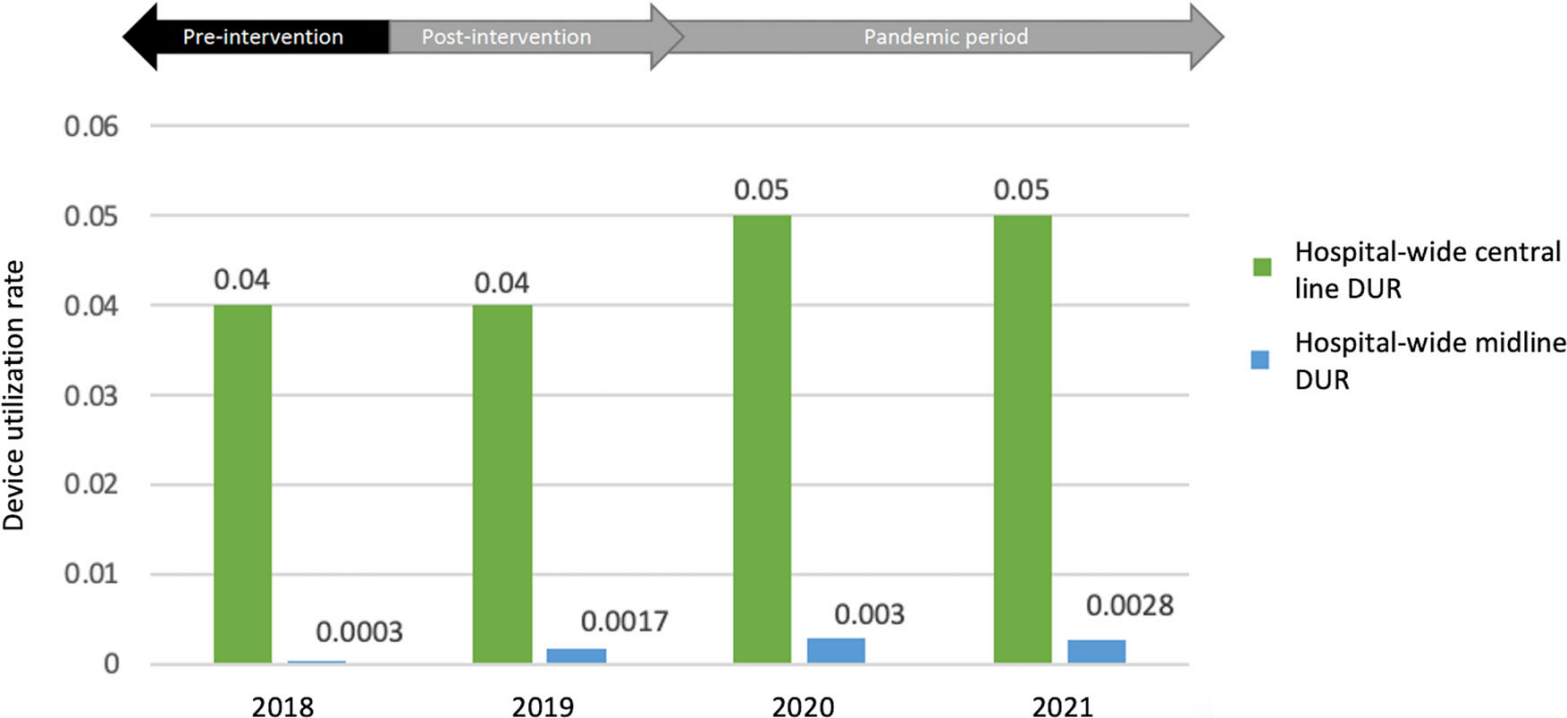
Hospital-wide device utilization rates for central lines and midlines during our preintervention and postintervention period

In the preintervention period, 63 peripherally inserted lines were placed, with 55 (87%) PICCs and 8 (13%) MLs (Figure 1). In the first year following the intervention, 76 lines were placed, 48 (63%) of which were MLs. This trend was sustained throughout the COVID-19 pandemic, with 116 lines placed in 2020 (80% ML) and 96 lines in 2021 (88% ML). Hospital-wide DUR per 1000 patient days for CLs (CVCs and PICCs) changed from 0.04 in 2018 and 2019 to 0.05 in 2020 and 2021 (Figure 2). Hospital-wide ML DUR changed from 0.0003 in 2018 to 0.0017, 0.003 and 0.0028 in 2019, 2020, and 2021, respectively (Figure 2). All ML recommendations were accepted by providers.

### BSIs, DVTs, and cost

No BSIs were reported during the preintervention period and the first year postintervention. During the COVID-19 pandemic, 8 BSIs were associated with MLs and 3 with PICCs. The most common causal organisms were *Candida* (37%) and *Enterococcus* (27%), followed by *Enterobacter* (9%), *S. aureus* (9%), and *Pseudomonas* (9%). Most (72%) BSIs were in COVID-19 cases and all (100%) BSIs had secondary sources of infection unrelated to CLs. All ML BSIs would have met NHSN criteria for CLABSI if a CL had been present, corresponding to the potential avoidance of 8 CLABSIs and estimated cost savings of $224,864. No DVTs were detected during the study period.

## Conclusion

ML prioritization was successfully implemented at our safety-net hospital and sustained throughout the COVID-19 pandemic. Our program resulted in a significant increase in ML utilization and a decline in PICC use from 87% to 12%, suggesting that PICCs were previously used for venous access without CL indications or remained after those indications were no longer present.

High acuity during the pandemic likely contributed to an increase in DUR and BSIs. Case audits revealed these BSIs would have fulfilled NHSN criteria for CLABSIs despite having secondary sources identified. The BSIs were unrelated to venous access and were likely unavoidable. NSHN surveillance definitions may not always align with clinical judgment or claims-based CLABSI indicators, allowing for BSIs with secondary sources to be classified as CLABSIs with financial consequences to hospitals and patients.^8^ Studies have shown lower BSI rates with MLs. A meta-analysis of 20 studies revealed BSI risks of 0.4% for ML and 2.4% for PICCs.^9^ In addition to these safety benefits, ML prioritization may carry cost advantages. MLs are nurse-inserted, do not require postplacement imaging, and have fewer insertion complications that can prolong hospitalizations, such as bleeding or pneumothorax. The cost of ML insertion is approximately $2,000 lower than PICCs.^10^ BSIs occurring with MLs may incur less direct and indirect costs compared to CLABSIs.^6,7^ Though costs may vary by insurance, the estimated cost of a CLABSI is approximately $28,108 higher than BSIs and there are additional indirect savings by avoiding CLABSI-related pay-for-performance payer penalties. The comparative risk of venous thrombi between ML and PICCs is uncertain, with some studies showing higher risk of superficial venous thrombosis with MLs but less risk of DVTs. Further studies are needed to better clarify this risk.

Our study has many limitations, including its quasi-experimental single-center design that may limit the generalizability of our findings. The study was conducted during the COVID-19 pandemic, which may have introduced confounding factors, such as the increase in BSIs during this period. Future research should investigate the long-term effects of ML prioritization in a variety of healthcare settings and explore the potential benefits of this approach beyond infection prevention and cost savings. This may include evaluating the impact on patient satisfaction, healthcare provider workload, and overall healthcare system efficiency.

We report the successful implementation of a ML prioritization initiative at a safety-net community hospital and its sustainability despite the COVID-19 pandemic. The reduction in PICC use and the potential avoidance of CLABSIs suggests the potential to improve patient outcomes and generate cost savings.

## Supporting information

Mena Lora et al. supplementary material Mena Lora et al. supplementary material

## Supplementary material

For supplementary material accompanying this paper visit http://doi.org/10.1017/ash.2024.21. click here to view supplementary material
