# Supplementary material for: Impact of a midline catheter prioritization initiative on device utilization and central line-associated bloodstream infections at an urban safety-net community hospital
Source: Antimicrob Steward Healthc Epidemiol. 2024 Feb 16;4(1):e27. doi: 10.1017/ash.2024.21 (PMC10897721; doi:10.1017/ash.2024.21)
Supplement: Mena Lora et al. supplementary material [file S2732494X24000214sup001.doc]

**SUPPLEMENT A.** Midline education to RNs and Physicians

**Purpose:** To prevent the risk of infections associated with central venous access devices, midlines will be used when venous access is needed and peripheral IV access is not feasible.

**Level of Responsibility**: RN, MD

**Definition:**CDC defines a midline catheter as a peripheral IV catheter, as distinct from a central venous catheter, measuring between 3 and 8 inches in length.

**Insertion Site:** The most favorable site for midline insertion is at the mid‐biceps level of the upper arm (i.e., the middle 1/3rd) in either the basilic or cephalic vein. The brachial vein may be used if deemed appropriate by the clinician.

**Contraindications to Midline Use:**

A midline catheter should not be used for the following indications:

**•** Continuous vesicant therapy

• Total parenteral nutrition (TPN)

• Solutions greater than 900 mOsm/L

• Vasopressors or caustic medications

• All infusates requiring central venous access (CVC)

For the above indications, a central venous catheter or PICC should be used.

**Daily monitoring and process**

**•** Central lines and PICC lines will be reviewed by infection prevention and the PICC team daily to assess if indications for CVC access are present.

**•** New orders for PICC lines or CVCs will be reviewed by infection prevention and the PICC team daily to assess if indications for CVC access are present.

**•** CVCs or PICCs will be used if any of these indications are met:

**•** Continuous vesicant therapy

• Total parenteral nutrition (TPN)

• Solutions greater than 900 mOsm/L

• Vasopressors or caustic medications

• All infusates requiring central venous access (CVC)

**•** If these indications are not met and peripheral IV access is not feasible, midline placement will be recommended to the ordering provider

**Important**: Midlines are cleared by FDA for less than 30 day usage; however, the CDC guidelines

allow for longer dwell times in excess of 30 days (<45 days), provided there is no evidence of infection or other complication.
